# Sero-prevalence of lumpy skin disease in selected districts of West Wollega zone, Ethiopia

**DOI:** 10.1186/s12917-015-0432-7

**Published:** 2015-06-17

**Authors:** Zelalem Abera, Hailu Degefu, Getachew Gari, Menbere Kidane

**Affiliations:** College of Medical and Health Sciences, School of Veterinary Medicine, Wollega University, P. O. Box 395, Nekemte, Ethiopia; Colleges of Agriculture and Veterinary Medicine, Head School of Veterinary Medicine, Jimma University, P. O. Box 307, Jimma, Ethiopia; National Animal Health Diagnostic and Investigation Center (NAHDIC), P. O. Box 04, Sebeta, Ethiopia

**Keywords:** Cattle, Ethiopia, LSD, Risk factors, Seroprevalence, West Wollega

## Abstract

**Background:**

Lumpy skin disease (LSD) is an economically devastating emerging viral disease of cattle caused by a virus associated with the Neethlig poxvirus in the genus *Capripoxvirus* of the family *Poxviridae.* A cross-sectional study was conducted from October, 2012 to May, 2013 in two districts of Western Wollega of Oromiya Regional State, with the objectives to determine animal and herd level seroprevalence of lumpy skin disease in the study area. The study population comprised of indigenous and crossbred cattle. Multi-stage sampling method was applied to select cattle and herd owners for the interviews. A total of 544 sera samples were collected from 252 herds and the serological test were conducted using indirect fluorescent antibody test (IFAT).

**Result:**

An overall individual level sero-prevalence of 6.43 % (n = 35) and herd level seroprevalence of 5.95 % (n = 15) were estimated. There was significant variation (P < 0.05) between the seroprevalence in Gimbi (4.41 %) and Lalo Assabi (8.46 %) districts at animal level. The sero- prevalence of LSD exposure among breeds (local and cross) was significantly different in that it was found significantly higher in cross breeds (OR = 2.85, p = 0.016) than in local zebu. There was statistically significant difference (p = 0.384) among the age groups (adult, young and calf) in the sero-prevalence of LSD. The average sero-prevalence according to age groups was 8.78 %, 5 % and 2.74 % in adults, youngs and calves, respectively and this shows the prevalence was very low in calves. The current finding revealed no significant variation between male and female animals (p > 0.05). In addition, there was no significant association between seropositivity to LSD and, the agro-climatic zones (midland and highland).

**Conclusion:**

The present study revealed a moderate distribution of sero-positive cattle in the study area and the disease observed warrants future detailed study on the spread of the disease in the area.

**Electronic supplementary material:**

The online version of this article (doi:10.1186/s12917-015-0432-7) contains supplementary material, which is available to authorized users.

## Background

The livestock sector globally is highly dynamic, and contributes 40 % of the global value of agricultural output, and supports the livelihoods and food security of almost a billion people [1]. In many developing countries, livestock keeping is a multifunctional activity. In addtion to their direct role in generating food and income, livestock are a valuable asset, serving as a store of wealth, collateral for credit and an essential safety net during times of crisis [2, 3].

**Fig. 1:**
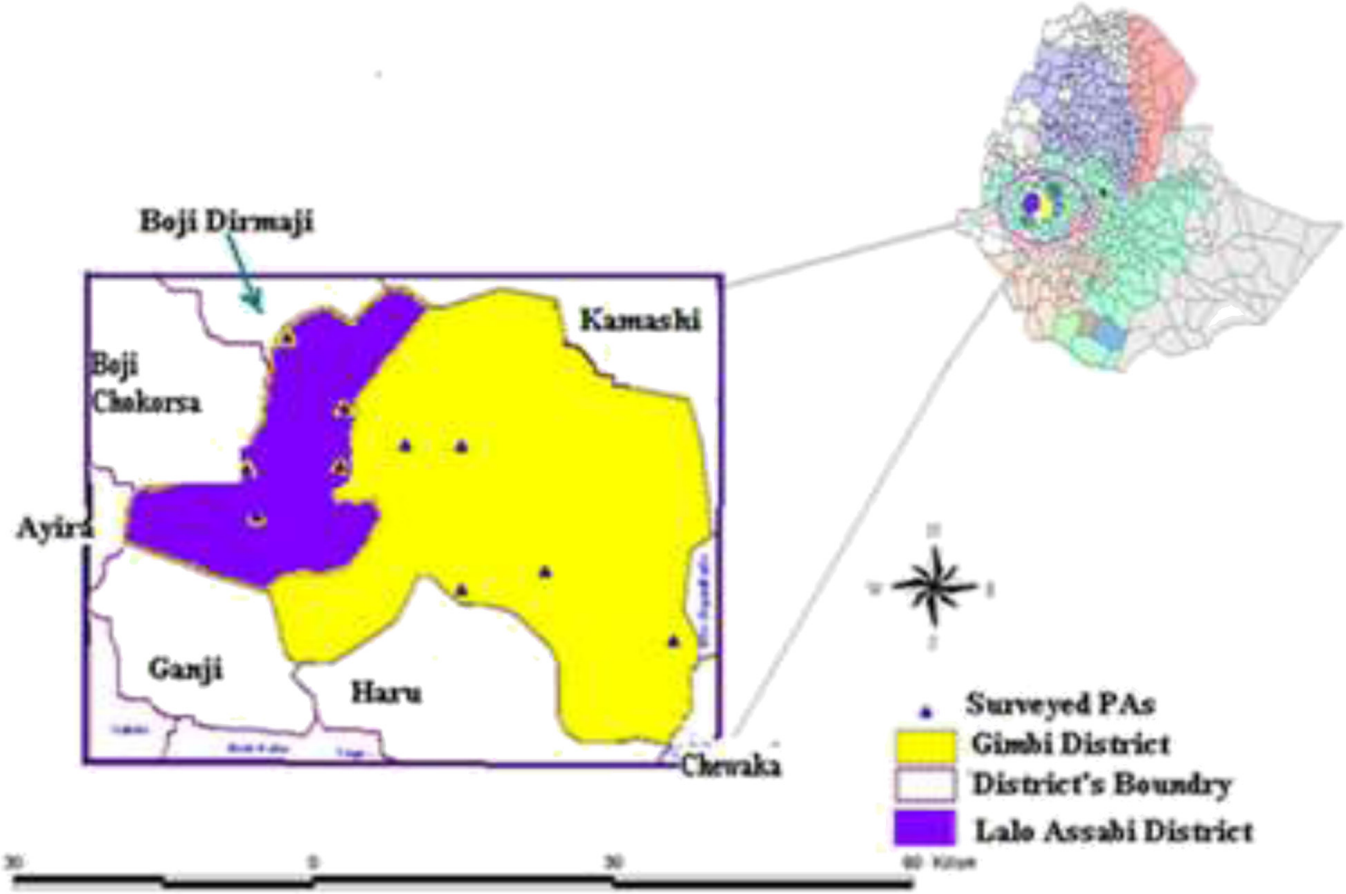
Map of the study Area

In Ethiopia livestock production is an integral part of the agricultural system contributing 40 % of the agricultural gross domestic product (GDP) and 20 % of the total GDP without considering other contributions like provision of traction power, organic fertilizers and as means of transport [4, 5].

In future, livestock production will increasingly be affected by competition for natural resources, particularly land and water, competition between food and feed and by the need to operate in a carbon-constrained economy [6]. Currently the overall livestock production constraints in Ethiopia are feed and water shortages, livestock diseases, low genetic potential of indigenous livestock and lack of marketing infrastructure [7, 8]. Lumpy skin disease is one of the many other diseases, which are known in causing economic losses and poor productivity in livestock [9–11]. Lumpy skin disease (LSD) is a generalized skin disease which is an infectious, eruptive and occasionally fatal disease of cattle caused by a virus associated with the neethlig poxvirus in the genus *Capripoxvirus* of the family *Poxviridae* [12–15].

The economic losses due to this disease is associated with decreased milk production, loss of traction power, weight loss, poor growth, abortion, infertility and skin damage. Pneumonia is a common sequel in animals with lesions in the mouth and respiratory tract [16–21].

LSD was first observed in the western part of Ethiopia (southwest of Lake Tana) in 1983 [22]. It has now spread to almost all the regions and agro ecological zones [23, 9]. Some epidemiological studies have been carried out since the disease was established in the country, with the diverse agro-ecological and production systems [9].

Study based on seroprevalence in southern Ethiopia reported a prevalence of 6 % [24]. Targeted sampling from outbreak areas around Southern Range land, Wolliso town and north Ethiopia reported prevalences of 11.6 %, 27.9 % and 28 %, respectively [24–26]. A recently prevalence study [11] results showed higher herd prevalence recorded in Afar (51 %) and Tigray (37 %) regions.

Published information on the factors that influence the occurrence of LSD are not many however some studies indicated that LSD is a disease which affect all age group, in Africa imported *Bos Taurus* appear to be more susceptible than the indigenous breeds [16]. The LDSV was found to be associated with other viruses belonging to the genus Capripoxvirus [27].

A clinical case of LSD has been reported in other animals such as the Asian water buffalo from Egypt [28], while antibodies have been demonstrated in black and blue wild beests, Elan, Giraffe, greater Kudu and other animals species [29, 30].

Lumpy skin disease is a disease caused by a virus which is believed to be mainly transmitted by flying insects [12, 31–33]. Recently, [34] reported the potential role of ixodic tick in the transmission of LSDV. Weather changes such as cold may adversely affect the insect vector and infected saliva may contribute to the spread of the disease [35].

However, there is a gap in the epidemiological patterns of transboundary diseases particularly lumpy skin disease in West Wollega zone except for a few outbreak reports. The study area interfaces with the pastoralists often crossing the border to other African courtiers (Sudan and South Sudan) and Benishangul Gumuz Regional State of Ethiopia. Therefore, the objective of this research was to determine animal and herd level sero-prevalence of lumpy skin disease in the study area.

## Materials and Methods

### Description of study areas

The study was conducted in two selected districts (Gimbi and Lalo Asabi) of West Wollega Zone of Oromiya Regional State; Western Ethiopia. West Wollega is one of the 18 Administrative Zones of Oromiya National Regional State. Administratively, the Zone has 21 districts, 19 of which are rural districts and 2 are urban administrations which are again subdivided into 533 kebele administrative units (487 rural and 46 urban Peasant Associations). Gimbi Town, which is located at a distance of 441 km from Addis Ababa, is the capital of the Zone, it is located between 8° 12'–10° 03' N and 34° 08'–36° 10' E. The Zone shares borderes with Benishangul-Gumuz Regional State, Qellem Wollega Zone, East Wollega Zone, Illubabor Zone and Gambella Regional State in the Northwest, Northeast and east; West, East, and in the South directions, respectively. The land area of the Zone is estimated to be 14,160.29 km^2^. ‘It experiences a tropical climate with relatively high mean annual temperature of 15 °C to over 25 °C [36] Fig. 1.

The annual rainfall pattern in the Zone decreases from East to West following the physiographic nature of the Zone. The mean annual rainfall of the Eastern high land ranges from 1800–2000 mm, while in the central plateaus, it ranges between 1600–1800 mm and in the remaining parts of the Zone it becomes between 1200–1600 mm. In the South-western parts of the Zone, it is even less than 1200 mm [36].

Livestock population of West Wollega Zone is 1,775,404 cattle, 385,098 Sheep, 353,385 Caprine, 137,926 (Donkey, Horse and Mule), 2,066,678 Poultry and 620,397 Bee colonies [37]. The farming system in the zone is mixed (Livestock production integrated with crop Production). Livestock production system is usually extensive, and the most common breeds are the local zebu breeds. Common grasslands provide extensive pasture for all parts of the areas of both study districts.

The study was conducted in Gimbi district which located between 9°–17° N and 35°–36° E and at altitudinal range of 1200 m–2222 m above sea level (masl). It has the mean minimum and maximum annual temperature ranges between 10 °C and 30 °C, respectively. The mean annual rainfall is 1400–1800 ml. It lies as reported by [38] and in Lalo Asabi district which lies between 9°–20° N and 35°–45° E and located in the Eastern part of West Wellega Zone. Enango town is its capital which is 23 km far away from the Capital of the Zone (Gimbi). It has an area of 43516 ha land, 185 ml monthly average rainfall and Altitudinal ranges between 1800 and 2200 masl. It shares common boundaries with Gimbi, Guliso, Bodji and Yubdo districts, and Benishangul-Gumuz Regional State [39].

### Study population

Animals involved in this study were all indigenous zebu and crossbred cattle population of all age groups above six months (>6 month). The districts were purposively selected based on the accessibility, lack of sero-prevalence information, presence of livestock markets activity, production and management system, history of contact with wild life and transboundary animal’s movement from other pastoralist area of neighbouring Regional States of Ethiopia. These districts share similar farming system but different in agrological locations. They have also different ranges of livestock population (Table 1).

**Table 1:** Summary of Peasant Associations, herd owners and livestock population in both districts

Name of districts	N^o^ of PAs in districts			Name of selected PAs	Total herd owners	N^o^ of selected herd owners	Cattle Pop^n^ in PA	Sample size in PAs	Altitude
	Rural	Urban	Total						
Gimbi	31	1	32	Were Seyo	150	23	1,725	63	1903
				Bikiltu Tokuma	460	28	2,734	58	1821
				Jogir	579	25	2,422	46	1298
				Chutta Kaki	421	23	2,474	44	2016
				Lelisa Yesus	510	28	2,248	61	1851
Subtotal	31	1	32	5	2,120	127	11,603	272	-
Lalo Assabi	27	4	31	Horda Daleti	707	25	994	48	1766
				Nebo Daleti	513	26	1,040	57	1618
				Werebabo Siben	620	23	1,212	52	1933
				Haroji Serdo	652	28	1,344	60	1936
				Dongoro Dissi	416	23	1,724	55	1937
Subtotal	27	4	31	5	2,908	125	6,314	272	-
G. total	58	5	63	10	5,028	252	17,917	544	

N^o^: Number; Pop^n^: Population.

### Study design

A cross-sectional study was carried out from October 2012-May 2013 to determine the sero-prevalence of Lumpy skin disease in the study area. Multi-stage sampling method was followed to select the sampling units and districts, Peasant Associations (PAs), herds and animals were selected to be included in the study. Animals included in the study were distributed over the selected districts. Five PAs were randomly selected from each district in consultation with the respective district Agricultural Office; especially Livestock Resource, Development and Health Agency expert’s based on location and accessibility.

From selected PA’s, a herd was selected as a primary epidemiological unit, and by assuming an average number of 10 animals per herd; a total of 252 herds were randomly selected. In each PA, the number of selected herds range from 22 (Were Seyo of Gimbi District) to 28 (Bikiltu Tokuma of Gimbi and Dongoro Dissi of Lalo Assabi Districts) in both districts. The term “herd” mean a cluster or aggregate of animals’ those have similar resource of feeding, drinking and etc. Additionally, the extensive management system implies that animals from the same Peasant Associations share communal grazing and watering resources and experience the same environmental and climatic conditions.

A range of one to three (1–3) cattle from the selected herds and fourty four to sixty three (44–63) animals from each PA were randomly selected to be included in the study based on the representativeness of the Peasant Associations (PAs) and districts. During sample collection, the estimated age of each sampled animal was determined by consulting the owners of the cattle. The sampled animals were categorized as calves (>0.5-2years), young (>2– 4 years) and adults (>4 years) [42].

### Sampling technique and sample size determination

The simple random sampling technique was followed, to select individual animals to be used for the study in the study area.

Minimum sample size for this cross-sectional study was calculated using the formula by [40, 41] with 95 % confidence level and 5 % absolute precision.

The sample size was achieved by assuming the sero-prevalence to be at the expected of Lumpy Skin Disease (LSD) at animal level (23 %) [9]. Accordingly, 272 desired sample sizes for the study were calculated.

$$ \mathrm{n}=\frac{1.96^2 Pexp\left(1-\mathrm{Pexp}\right)}{d^2} $$

Where: n = required sample size;

exp = expected prevalence;

d = desired absolute precision.

### Serum sample collection, submission and processing

#### Serum sample collection and handling

Full disposable 10 ml sterile vacutainer tubes of whole blood samples were collected from the jugular vein of each animal. The tubes were then kept protected from direct sun light at room temperature in slant position until the blood clotted and sera were separated within 12 h. The separated sera were transferred to sterile cryovials; bearing the names of PAs, animal number, age and sex and kept in icebox at the field (Additional file 1).

Finally, the samples were transported to the National Animal Health Diagnostic and Investigation Centre (NAHDIC), Sebeta, for serological examination using Indirect Fluorescent Antibody Test (IFAT). In the laboratory; the sera were preserved at-20 °C until laboratory investigation [43]. Additionally, test principles and test procedures for IFAT set by Hemagen Diagnostics Inc, were used (Additional file 2) and an evaluation of Indirect Fluorescent Antibody Test done by [44] demonstrated that IFAT has a reasonable high accuracy to be used for the diagnosis and sero-surveillance analysis of LSD.

### Procedures of the test

The procedures of the IFAT were essentially as described by [44] are carried out in two basic reaction steps. Procedurally four main points were listed. These were cell seeding, cell infection and fixation and testing of sera (Additional file 3).

### Data management and analysis

Data entry and management was made using Microsoft Excel sheets. Data analysis was made using Statistical Package for Social Science (SPSS 2007, version 16) software.

The Odds ratio was calculated for each risk factor for sero-positivity to LSD. In all the analyses, confidence levels at 95 % were calculated, and a P < 0.05 was used for statistical significance level. The odds ratio (OR) was calculated for the risk factors and sero-positivity of the disease to determine the degree of association risk factors and the disease. Descriptive statistics like prevalence was used to calculate sero-positivity by dividing the number of LSD positive animals by the total number of animals tested and the herd prevalence was determined by dividing positive herds to total number of herds and the herd would be considered positive if one or more animal in the herd would be positive to lumpy skin disease.

### Ethical considerations

All the farmers who participated consented to the study. The purpose of the study was explained well to the participants, etc. Also assure them of the confidentiality of results and we have showed a polite behaviour to make them active participant in the study.

## Results

### Animal level sero-prevalence

The overall sero prevalence of lumpy skin disease in the study area was 6.43 % (*p* = 0.05, OR = 2, 95 % CI =5.43-12.41). Between the two districts included in the study, the sero prevalence was significantly higher in Lalo Assabi animals as compared to animals from Gimbi District (Table 2).

**Table 2:** Sero-prevalence of Lumpy Skin Disease in Gimbi and Lalo Assabi districts of West Wollega Zone

District	Animal tested	No of Sero Positive (%)	P-value	OR (95 % CI)	95 % CI
Gimbi	272	12 (4.41)			2.30–7.52
Lalo Assabi	272	23 (8.46)	0.05	2(0.90–8.03)	5.43–12.41
Total	544	35 (6.43)			4.52–8.83

### Herd level sero-prevalence

Among the 252 herds investigated in this study, 15 (95 % CI = 3.38–9.66) of the herds had at least one positive using IFAT for LSD. In this study, herd-level risk factors were considered and examined by logistic regression for presence of any association with herd-level sero positivity to Lumpy skin disease virus. Except breed of animals and Peasant Association, none of the risk factors considered in the analysis had significant effect on herd-level sero prevalence to LSD (Table 3).

**Table 3:** Sero-prevalence of LSD at the herd level in Gimbi and Lalo Assabi districts

District	No of examined Herds	No of positive Herds (%)	P-value	OR (95 % CI)	95 % CI
Gimbi	127	4 (4.15)	-	-	-
Lalo Assabi	125	11 (8.8)	0.069	0.337	3.38-9.66
Total	252	15 (5.95)	0.035		

On the other hand, there was variation in the sero-prevalence of LSD occurrence among the cattle of different Kebeles selected for the study. Relatively high seroprevalence records were observed in Dongoro Dissi (15 %). On the contrary, all sera samples taken from Jogir and Were Seyo showed zero positivity for IFAT test we used in this study (Table 4).

**Table 4:** Descriptive and Analytic Results of Sero prevalence of LSD for Cattle of different Kebeles

PAs in both districts	No of sampled	No of Sero Positive (%)	P-value	OR (95 % CI)	95 % CI
Were Seyo	63	0 (0)	0.363	-	-
BikiltuTokuma^a^	58	1 (1.72)	0.031	-	0.04–9.23
Jogir	46	0 (0)	-	-	-
Chutta Kaki	44	3 (6.81)	0.004^b^	0.2	1.42–28.65
LelisaYesus	61	8 (13.11)	0.023^b^	8.6	5.83–27.22
Horda Daleti	48	1 (2.12)	0.208	1.2	0.05–11.07
Nebo Daleti	57	5 (8.77)	0.766	5.4	2.91–19.29
Werebabo Siben	52	3 (5.77)	0.049^b^	4.4	1.29–15.94
Haroji Serdo	55	5 (9.1)	0.305	5.1	3.01–19.93
Dongoro Dissi	60	9 (15)	0.003^b^	10	7.10–26.57
Ground Total	544	35 (6.43)	0.175	3.49	

^a^Reference variable for OR

^b^Statistical significance

### Sero-prevalence of LSD based on sex, age, breed and altitude differences

The sero-prevalence between female and male animals was studied and out of animals sampled, the majority or 64.0 % were females while about 36.0 % of them were males. The sero-prevalences were 7.65 and 5.74 % in female and male, respectively (Table 5). However, there was no statistical difference between the two sexes.

**Table 5:** Sero-prevalence of Lumpy Skin Disease According to Sex, Age, Breed and Altitude differences in the area

Risk factors	Animal tested	Number of positive (%)	P-value	OR (95 %)	95 % CI
Age					
Adult	251	22 (8.78)	0.097	3.41 (0.8–30.3)	5.56–12.96
Young	220	11 (5)	-	1.86 (0.3–17.7)	2.52–8.77
Calves^a^	73	2 (2.74)	-	-	0.3 – 9.54
Sex					
Male	348	20 (5.74)	-		3.54 – 8.73
Female	196	15 (7.65)	0.384		4.34 – 12.31
Breed					
Local^a^	496	28 (5.61)	-	-	2.3–19.8
Cross	48	7 (14.58)	0.016	2.85 (1.2–6.9)^b^	3.7–8.77
Altitude					
Highland	274	20 (7.23)			4.51–11.11
Midland	270	15 (5.55)	0.41	1.3 ( 0.63–2.91)	3.31–8.90
Ground Total	544	35 (6.43)			

^a^Reference variable for OR

^b^Statistical significance

Analysis of age wise prevalence of Lumpy Skin Disease indicated that the difference in prevalence among the three age groups were relatively high in adult group (Table 5) than the young and calf age groups with no statistically significant variation.

The breed of animals showed a significant association with LSD seropositivity with cross breed being approximately 3 times more likely to be seropositive compared to indigenous animals (OR = 2.85; 95%CI: 1.2–6.9, *P* = 0.016). Based on altitude differences the target area was broadly classified into midland or ‘Weynadega’ (1200–1900 m) and highland or ‘Dega’ (>1900 m). Thus, comparison was made on the sero-prevalences of the Highland (‘Dega’) having 9.48 % and Midland (‘Weynadega’) with 5.55 % (Table 5). There was no significant variation in sero-prevalence between the 2 agro climates at individual level.

## Discussion

In the present study, Lumpy Skin Disease Virus (LSDv) exposure was investigated in the two administrative districts of West Wollega Zone (Gimbi and Lalo-Assabi) by applying field study, serological analysis.

### Animal level sero-prevalence and associated risk factors

The 6.43 % seroprevalence of Lumpy Skin Disease recorded in cattle of the study was close to the animal level (6 %) and overall (8.1 %) sero prevalences recorded by [24] in southern Ethiopia and [9] for the different agro-ecological zones in Ethiopia. It is worth mentioning, other studies based on clinical observation on the disease were made around Nekemet which is close to this study area and, 7 % prevalence was reported [43]. Again, targeted study on outbreak areas of Southern Range land, around Wolliso town and in three districts of eastern Amhara region reported prevalence of 11.6, 27.9 and 28 %; respectively [24-26].

In the present investigation, the overall animal sero-prevalences of LSD (6.43 %) in the two administrative districts of West Wollega namely Gimbi (4.41 %) and Lalo-Assabi (8.46 %) showed a significant variation (*p* = 0.05, OR = 2, 95 % CI =5.43-12.41) with logistic regression analysis. Similarly the overall prevalence observed in Lelisa Yesus (13.11 %) and Dongoro Dissi (15 %) was significantly high as compared to the rest of the Kebeles which was due to factors like sharing common boundary with Beni-Shangul Gumuz Regional state, focal grazing point and high livestock trade activity.

This finding agrees well with the finding of [24], who stated a difference in the frequency of occurrence of LSD across 15 districts they selected for their study. In addition, many factors such as season, insect vector activity, the health status and breed of the animals can affect the magnitude and the occurrence of LSD [23, 30, 31, 45].

In the present study, an attempt has been made to compare the susceptibility of the indigenous (Zebu) and crossbred (Zebu x Frisian) breeds of cattle raised in the same management system. The result revealed a significantly higher sero-postivity result in the cross breed (OR = 2.85, 95%CI: 1.2– 6.9, *P* = 0.016).). This result some how goes with the previously suggested idea that, the breeds of *Bos taurus*, imported into Africa from Europe, or Australia are far more susceptible than the indigenous *Bos indicus* cattle [16,23,30].

Analysis of the association between age and sero-positivity for LSD revealed no statistically significant variation among the three age categories; however, the sero prevalence in calves is very low as compared to adult and young age groups. This may be indicative of prevailing passive maternal immunity and low frequency of exposure.

Similar to this finding [46] reported that, suckling calves showed the lowest attack rate, though in the dynamic model younger cattle did not show higher susceptibility to infection in their study of mathematical modelling and evaluation of the different routes of transmission of lumpy skin disease virus during a certain outbreak. There were no previous reports of age related susceptibility to LSD. A possible alternative explanation for the lower sero prevalence recorded in calves in this study may be associated with lower susceptibility of calves to biting by flies as previously described [47]. Another potential explanation can be associated with location, as the lowest prevalence was documented in the calves, which were kept at homestead where there is less insect vector activity. The study revealed high sero prevalence (8.78 %) in adults, in which the maternal immunity level drops and exposed to diseases, as the age increases.

The absence of significant association (p > 0.05) between sex and sero positivity to LSD was observed in current investigation using bivariate analysis, but [48] reported that lactating cows seem to be the most susceptible. On the contrary, [49] indicated that, male zebu cattle had higher cumulative incidence than females and this might be attributable to the stress factor of exhaustion and fatigue rather than to a biological reason. Another reason given by [9] also mentioned that, the majority of male animals were draft oxen used for heavy labour, which might contribute to an increase in susceptibility. The same authors also reported as draft oxen cannot protect themselves well from biting flies when harnessed in the yolk, and the beat scratches on their skin induced while ploughing may attract biting flies potentially capable of transmitting LSD infection.

Relatively higher sero-prevalences were found in the highland (7.23 %) than midland (5.55 %) with no statistical variation in this study. On the other hand, [24] found out that LSD occurrence to be high in midland and lowland agro-climates than the highland agro-climate in some other parts of Ethiopia. In addition, a recent study done by the same authors in 2012 based on serology estimated by using a Bayesian model and herd level sero-prevalence was higher in the midland (64 %) as compared to the lowland (50 %) and the highland (26 %) agro-climatic Zones of Ethiopia.

### Herd level sero prevalence and associated risk factors

The overall herd prevalence recorded in this study (5.97 %) was very low when compared to the previous herd level reports of 64, 26 and 50 % for midland, highland and low land agro climate zones of Ethiopia [10]. But, the presence of a single sero positive herd could also be in support of herd level endemicity of LSD in the area. However, none of the factors considered for herd-level prevalence in the study were significant, the influences of management related risk factors and characteristics of the population for occurrence of infection in a herd are reported to have an important role [9,10].

## Conclusion and recommendations

The present cross-sectional study indicated that lumpy skin disease is an important disease in the western Wollega zone of Oromia regional state of Ethiopia. Even if the recorded sero-prevalence is moderate, the disease is found to be spreading in to new areas that have been considered

previously as free areas (Peasant Associations or districts) of the zone and would be a major livestock health problem. Therefore, the use of mass vaccination applied to all breeds of cattle in both districts using an effective vaccine against LSD, such as the attenuated Neethling strain vaccine need to be considered.

Further research is needed to assess the status of the disease and to suggest implementation of appropriate control and prevention methods in the areas. This study provides the preliminary information of the presence of LSDV infection in the West Wollega. This finding also gives attention on the distribution of LSDV in the study area and can assist planners, decision-makers; practitioners and researchers in their efforts. Also it could help them in disease surveillance and control activities for risk mitigation and to improve the health of animals.

## Additional files

Additional file 1:
**Reagents and materials required for the test.** Details of Reagents and materials required for the test during the study. Additional file 2:
**Principles of the test.** Principles of the test during the study were discussed in detail. Additional file 3:
**Indirect Fluorescent Antibody Test Laboratory Protocol.** Details of preparation of Ag coated Micro-plates and procedure to test the sera.

## Abbreviations

CI: Confidence interval
FITC: Fluorescence isothiocyanate
GDP: Gross Domestic Product
GG: *Getachew Gari*
HD: *Hailu Degefu*
IFAT: Indirect fluorescent antibody test
LDSV: Lumpy skin disease virus
LSD: Lumpy skin disease
MK: *Menbere Kidane*
NAHDIC: National animal health diagnostic and investigation centre
OR: Odd ratio
PA: Peasant association
PBS: Phosphate-buffered saline
PH: Power of hydrogen
SGPV: Sheep and goat pox virus
SPSS: Statistical package for social science
ZA: *Zelalem Abera*

## References

[1] Thornton PK (2010). Livestock production: recent trends, future prospects. The Royal Society.

[2] MoA (2006). Ministry of Agriculture and Rural Development, the Status of Animal Health Services in Ethiopia.

[3] FAO (2009). Livestock in balance.

[4] Aklilu Y, Irungu P, Alemayehu R (2003). An Audit of the Livestock Marketing Status in Kenya, Ethiopia and Sudan. Issues and Proposed Measures, Vol. II.

[5] Gebreegziabhare B: An over view of the role of Ethiopian livestock in livelihood and Food safety. Ministry of Agriculture and Rural development of Ethiopia; Presente on dialogue on livestock, food security and sustainability, a side event on the session of 22nd COAGO, FAO, Rome 2010.

[6] Thornton PK (2006). Mapping climate vulnerability and poverty in Africa.

[7] Markos T (1999). Livestock Production Constraints in a M2-2 Sub-Agro ecological Zone with Special Reference to Goat Production.

[8] Alemayehu M: Country pasture/forage profiles 2009.

[9] Gari G, Waret-Szkuta A, Grosbois V, Jacquiet P, Roger F (2010). Risk factors associated with observed clinical lumpy skin disease in Ethiopia. Epidemiol Infect.

[10] Gari G, Grosbois V, Waret-Szkuta A, Babiuk S, Jacquiet P, Roger F (2012). Lumpy skin disease in Ethiopia: Seroprevalence study across different agro-climate zones. Acta Trop.

[11] Birhanu, Assessments of the risk factors and financial impacts of LSD in selected districts of Tigray and Afar Regional States, Northeastern Ethiopia. M.Sc. Thesis 2012 in press.

[12] Chihota CM, Rennie LF, Kitching RP, Mello PS (2003). Attempted mechanical transmission of lumpy skin disease virus by biting insects. Med Vet Entomol.

[13] Stram Y, Kuznetzova L, Rubinstein-Guni M, Fridgut O, Yadin H (2006). The recent Lumpy Skin Disease outbreak in Israel: A molecular prospect.

[14] Ahmed WM, Zaher KS (2008). Observations on lumpy skin disease in local Egyptian cows with emphasis on its impact on ovarian function. Afr J Microbiol Res.

[15] Gari G, Bonnet P, Roger F, Waret-Szkuta A (2011). Epidemiological aspects and financial impact of lumpy skin disease in Ethiopia. Prev Vet Med.

[16] Davies FG (1991). Lumpy skin disease of cattle: A growing problem in Africa and the Near East Veterinary Research Laboratories.

[17] Kassa B, Bisrat M, Asegedech S, Africa J (1998). Control of “Ekeke” Skin Defects in Sheep by Insecticides and Shearing.

[18] McDermott J, Randolph T, Staal S (1999). The economics of optimal health and productivity in smallholder livestock systems in developing countries. Rev Sci Tech Off Int Epiz.

[19] Yacob H, Nesanet B, Dinka Part A (2008). Prevalence of major skin diseases in cattle, sheep and goats at Adama Veterinary Clinic, Oromia regional state, Ethiopia. Revue Méd Vet.

[20] Ocaido M, Otim C P and Kakaire D: Impact of major diseases and vectors in smallholder cattle production systems in different agro-ecological zones and farming systems in Uganda. FVM, Makerere University, Kampala, Uganda. Livestock Research for Rural Development 2009, 21(9).

[21] OIE (2010). Terrestrial Manual of Lumpy Skin Disease.

[22] Mebratu GY, Kassa B, Fikre Y, Bethany B (1984). Observations on the outbreak of lumpy skin disease in Ethiopia. La Revue d’Elevage et de Médecine Vétérinaire des Pays Tropicaux.

[23] Babiuk S, Bowden TR, Dalman B, Parkyn G, Copps J (2008). Quantification of lumpy skin disease virus following experimental infection in cattle. Transbound Emerg Dis.

[24] Gari G, Biteau-Coroller F, LeGoff C, Caufour P, Roger F (2008). Evaluation of indirect fluorescent antibody test (IFAT) for the diagnosis and screening of lumpy skin disease using Bayesian method. Vet Microbiol.

[25] Asegid B (1991). Epidemiological Study of Major Skin Diseases of Cattle in Southern Range Lands.

[26] Beshahwured S (1991). Outbreak of Lumpy Skin Disease in and around Wolliso.

[27] Kitching RP, Bhat PP, Black DN (1989). The characterization of African strains of Capri poxviruses. Epidemiol Infect.

[28] Ali AA, Esmat M, Attia H, Selim A, Abdelhamid YM (1990). Clinical and pathological studies on lumpy skin disease in Egypt. Vet Res.

[29] Hedger RS, Hamblin C (1983). Neutralizing antibodies to lumpy skin disease virus in African wildlife. Comp Immunol Microbiol.

[30] Barnard B, Munz E, Dumbell K, Prozesky L (1994). Lumpy Skin Disease.

[31] Chihota CM, Rennie LF, Kitching RP, Mellor PS (2001). Mechanical transmission of lumpy skin disease virus by *Aedesaegypti* (Diptera;Culicidae). Epidemiol Infect.

[32] Carn VM, Kitching RP (1995). An investigation of possible routes of transmission of lumpyskin disease (Neethling) virus. Epidemiol Infect.

[33] Carn VM (1996). The role of dipterous insects in the mechanical transmission of animal viruses. Br Vet J.

[34] Tuppurainen ES, Stoltsz WH, Troskie M, Wallace DB, Oura CA, Mellor PS (2010). A potential role for ixodid (Hard) tick vectors in the transmission of lumpy skin disease virus in cattle. Transbound Emerg Dis.

[35] Haig DA (1957). Lumpy skin disease. Bull Epizoot Dis Afr.

[36] Socio-economic Abstract of Districts of West Wollega Zone 2008/09.

[37] West Wollega Zonal Livestock Development & Health Agency Office 2011.

[38] Ghimbi District Finance and Economic Development Office 2011.

[39] Socio-Economic Profile of Lalo Assabi District 2011.

[40] Thrusfield M (2007). Veterinary epidemiology.

[41] Thrusfield M (2005). Veterinary epidemiology.

[42] Berecha B, Gelagay A, Moses K, Yasmin J, Esayas G (2011). Study on seroprevalence, risk factors, and economic impact of foot-and-mouth disease in Borena pastoral and agro-pastoral system, southern Ethiopia. Tropl Anim Health Prod.

[43] Regassa C (2003). Preliminary study of major skin diseases of cattle coming to Nekemte Veterinary Clinic.

[44] Domenech J, Lubroth J, Eddi C, Martin, Roger F. Regional and international approaches on prevention and control of animal transboundary and emerging diseases. Ann. NY Acad. Sci. 2006;1081:90-107. 10.1196/annals.1373.010 17135498

[45] Kitching R, Taylor P (1985). Insect transmission of Capri poxviruses. Res Vet Sci.

[46] Rweyemamu M, Paskin R, Benkirane A, Martin V, Roeder P, Wojciechowski K, House JA, Kocan KM, Gibbs EPJ (2000). Emerging diseases of Africa and the Middle East. Tropical Veterinary Diseases - Control and Prevention in the Context of the New World Order.

[47] Troyo A, Calderon-Arguedas O, Fuller DO, Solano ME, Avendano A, Arheart KL (2008). Seasonal profiles of Aedes aegypti (Diptera:Culicidae) Larval habitats in an urban area of Costa Rica with the history of mosquito control. J Vector Ecol.

[48] Tuppurainen ESM, Oura CAL (2012). Review: lumpy skin disease: an emerging threat to Europe, the middle east and Asia. Tran boundary and Emerging Diseases.

[49] Blood D, Radostits O, Henderson J (1983). Veterinary Medicine.

