# Supplementary material for: Sero-prevalence of lumpy skin disease in selected districts of West Wollega zone, Ethiopia
Source: BMC Vet Res. 2015 Jun 17;11:135. doi: 10.1186/s12917-015-0432-7 (PMC4468805; doi:10.1186/s12917-015-0432-7)
Supplement: Additional file 1: — Reagents and materials required for the test. Details of Reagents and materials required for the test during the study. [file 12917_2015_432_MOESM1_ESM.docx]

**Additional File 2: Annex II. Reagents and materials required for the test**

**Reagents:** were Vero cells, viral suspension (Cpx vaccine, etc) properly diluted, cell medium MEM with EARLE salts; 10% fetal calf serum (FCS) that contain 1% de-glutamine in ml, cell medium MEM with EARLE salts that contain 1% de-glutamine in ml, L-glutamine, Fetal calf serum (Fcs), Trypsin-versene, Trypan blue, PBS1x, Ice, 70% Alcohol, 80% acetone, Anti-PPR monoclonal antibody, Anti-goat antibody conjugated with FITC, Dilution fluid (DF) (monoclonal antibody and conjugated) 1 % skimmed milk in PBS

**Materials:** were paper roll, Timer, Rotary microtiter plate shaker, Incubator (equipped with rotary microtiter plate shaker), Multichannel pipettor, Single channel pipettor, Glassic Pipettes (1ml, 5ml &13ml), Yellow tips, Troughs and Adhesive plate cover.
