# Supplementary material for: Sero-prevalence of lumpy skin disease in selected districts of West Wollega zone, Ethiopia
Source: BMC Vet Res. 2015 Jun 17;11:135. doi: 10.1186/s12917-015-0432-7 (PMC4468805; doi:10.1186/s12917-015-0432-7)
Supplement: Additional file 2: — Principles of the test. Principles of the test during the study were discussed in detail. [file 12917_2015_432_MOESM2_ESM.docx]

**Annexes**

**Additional File 1: Indirect Fluorescent Antibody Test Laboratory Protocol**

**Annex I: A. Preparation of Ag coated Microplates (96 wells Tissue grade Microplate)**

1. Prepare cell culture media (MEM +10% Fetal Calf Serum (FCS) + Antibiotics optional)

2. Add 100 μl of lamp testis cells suspension (2*104 cells/ml) to each 96 wells

3. Incubate the plates for 24hrs in 37°c and 5% CO2

4. Prepare the KS1 virus dilution of 2*103 TCID50 /ml in MEM cell media (without FCS)

5. Remove the previous media in the plate slowly using multichannel pipette keeping the plate in

gentle slope to remove all

6. Add 50 μl viral suspensions to each well and incubate at 37°c for 2hrs

7. Add 150 μl media with 10% Fetal Calf Serum to all wells and keep in 37°c for 48hrs

8. Remove the media by multi channel pipette in the same way as n° 5 to infect the cell

9. Add 100 μl acetone 80% cooled at 4°c to all wells for fixation and discard by pipette

10. Add 50 μl acetone 80% to each well & keep the plate in -20°c for 30min

11. Remove the acetone, wrap the plate with Aluminum paper and keep in -20°c until the plate is required for examination

**Annex I: B. Procedure to test the sera**

1. Add 50 μl PBS- 0.01M to each well and keep for 30min at room temperature.

2. Add 50μl blocking solution (1% skimmed milk or 0.5% lamb serum in 0.01M PBS) to each

well and incubate in 37°c for 20min

3. Dilute the test sera in blocking solution at 1/25 dilution

4. Take your record sheet and write the layout of the Microplate to add the test sera, strong +ve,

weak +ve and negative control sera.

5. Add 50 μl of diluted serum to the microplate in duplicate wells according to the layout and

incubate at 37°c for 30min

6. Rinse each well with 200 μl PBS 0.01M and discard by turning up-side down, repeat 3 times

7. Dilute the Fluorescein isothiocyanate conjugated anti-bovine gamma-globuline (IgG) of Rabbit

serum (FITC Rockland) in 1/40 dilution in blocking buffer solution

(Note: Antisheep and Anti-goat FITC from Dako also work perfectly like anti bovine FITC)

8. Add 50 μl diluted FITC to each well & keep in 37°c for 30min

9. (6) Wash the plate by 200 μl PBS 3 times

10. Add 50 μl PBS to each well and observe under UV light microscope.

**Additional File 2: Annex II. Reagents and materials required for the test**

**Reagents:** were Vero cells, viral suspension (Cpx vaccine, etc) properly diluted, cell medium MEM with EARLE salts; 10% fetal calf serum (FCS) that contain 1% de-glutamine in ml, cell medium MEM with EARLE salts that contain 1% de-glutamine in ml, L-glutamine, Fetal calf serum (Fcs), Trypsin-versene, Trypan blue, PBS1x, Ice, 70% Alcohol, 80% acetone, Anti-PPR monoclonal antibody, Anti-goat antibody conjugated with FITC, Dilution fluid (DF) (monoclonal antibody and conjugated) 1 % skimmed milk in PBS

**Materials:** were paper roll, Timer, Rotary microtiter plate shaker, Incubator (equipped with rotary microtiter plate shaker), Multichannel pipettor, Single channel pipettor, Glassic Pipettes (1ml, 5ml &13ml), Yellow tips, Troughs and Adhesive plate cover.

**Additional File 3: Annex III. Principles of the test**

The VIRGO fluorescent antibody assays utilize the indirect method of fluorescent antibody staining; first described by Weller and Coons in 1954. The procedure is carried out in two basic reaction steps. In step one, the serum to be tested is brought into contact with the antigenic substrate. Antibody, if present in the test serum, will attach to the antigen, forming an antigen-antibody complex. If the serum being tested does not contain antibody for this particular antigen, no complex is formed and all the serum components are washed away in the rinse step.

In the second step, the infected cells were fixed and allowed to react with Capri pox antibodies. In addition, it involves the adding of a fluorescein labelled antisheep and goat antibody to the test wells. If the specific antigen-antibody complex is formed in step one, the fluorescein labelled antibody will attach to the antibody moiety of the complex in step two.

The plates were read using Inverted fluorescence microscope under 40× magnifications. The positive tested serum appears with bright fluorescence foci where the antibody has reacted with the virus (apple green when stimulated by UV or blue light) and the negative serum appears as a dark field or dim gray foci.
